# Notch signaling pathway involved in *Echinococcus granulosus* infection regulates dendritic cell development and differentiation

**DOI:** 10.3389/fcimb.2023.1147025

**Published:** 2023-05-19

**Authors:** Mingxia Wang, Zailing Shang, Fei Qiao, Junhu Hei, Xueling Ma, Yana Wang

**Affiliations:** ^1^ Basic Medical Institute of Ningxia Medical University, Yinchuan, China; ^2^ Key Laboratory of Common Infectious Diseases of Ningxia Autonomous Region, Ningxia Medical University, Yinchuan, China

**Keywords:** Notch signal, dendritic cell, maturation, antigen, *Echinococcus granulosus*, immune response

## Abstract

**Introduction:**

The Notch signaling pathway is involved in the development of many diseases; it regulates the development of dendritic cells (DCs), and affects the immune response of DC-mediated T cells. We previously found that ferritin and malate dehydrogenase (mMDH) in Echinococcus granulosus (E.granulosus) induced different immune responses through sensitized DCs. Therefore, in the study we explored whether the Notch signaling pathway affects the development and differentiation of DCs, causing changes in the immune response of DCs sensitized with E. granulosus antigens, and clarified whether it is involved in E.granulosus infection.

**Methods:**

We used the Notch signaling pathway inhibitor [N-[3,5-difluorophenace-tyl] -L-alanyl]-S-phenylglycinet-butyl ester (DAPT) or activator Jagged1 to construct in vitro cell models with blocked or activated Notch signaling respectively. We analyzed the effect of Notch signaling on the development and differentiation of DCs by detecting their morphology, migration function, capacity to promote T cell proliferation, and cytokine secretion. We observed the changes in DC response to E. granulosus antigens and the mediated immune response.

**Results:**

DAPT inhibited the development and maturation of DCs, which were in a non-responsive or incompetent state, reduced the sensitization of DCs to Eg.ferritin, weakened the migration ability of DCs, disrupted their ability to mediate T-cell proliferation, reduced DC expression of MHCII, CD80, CD60, and CD40 co-stimulatory molecules, prevented the secretion of cytokines and attenuated the expression of Notch1, Notch2, Notch3 receptors, Jagged1, Delta-like 4 (Delta4), and Hes1. Following Jagged1 addition, the function of DCs was restored to some extent, and the expression of Notch1, Delta4 and Hes1 was activated in response to the stimulation of Eg.ferritin. However, Eg.mMDH stimulated DCs to produce an immune response showing weak interference by DAPT and Jagged1.

**Discussion:**

The study suggests that the Notc h signaling pathway is involved in the Eg.ferritin-sensitized DC-mediated immune response, which may become a new target for treating E.granulosus infection.

## 1 Introduction

The Notch signaling pathway is highly conserved in genetic evolution, and the Notch receptor family in mammals includes four receptors (Notch 1-4), five corresponding Notch ligands (Jagged1-2 and Delta-like1 [Delta-1], Delta-like3 [Delta-3], and Delta-4), all of which are widely expressed on the surface of various cells (Luca et al., 2017). As we all know Notch signaling have a wide and diverse regulatory role in cell fate during embryonic and postnatal development (Cheng et al., 2006; Ohlstein et al., 2015; Corson et al., 2017), and they recognize and regulate the innate and adaptive immune responses involved in the onset and development of infectious diseases (Shang et al., 2016), including bacterial (Schaller et al., 2016), viral (Ito et al., 2011), fungal (Jannuzzi et al., 2018), and parasitic diseases (Krawczyk et al., 2008). DCs as the first line of protection of the immune system in the body, play an important role in pathogen invasion. Notch signaling can be involved in the development of some parasitic diseases by regulating the development of DCs (Krawczyk et al., 2008) , thereby affecting the immune response induced by DCs. Research regarding cystic echinococcosis (CE) is limited. Ebrahim et al. found that Notch gene products were highly expressed in the germinal layer and vesicle/microcyst formation, and that they play a role in mitotic cell division and the proliferation of *E.granulosus* (Dezaki et al., 2016). However, whether the Notch signaling pathway is involved in *E. granulosu*s infection remains unclear.

CE is a chronic zoonotic disease caused by larvae of *E.granulosus* infecting an intermediate host (human or livestock). The disease has a global distribution, with approximately 5−30% of the people in western China having been exposed to *E. granulosus* (Zhang et al., 2015). It is highly severe, with current estimates of the global burden average being 285,500 with 5−7 disability-adjusted life years (DALYs) for patients with CE (Wen et al., 2019). CE has been listed as one of the 17 neglected diseases to be controlled and eliminated globally by 2050 (WHO, 2012). Therefore, the search for effective vaccine molecules is crucial (Wen et al., 2019).

We previously found that both ferritin and mMDH of *E. granulosus* play a crucial role in the life activity of *E.granulosus* and can be used as vaccine candidate molecules; however, the two antigens induce DCs to produce different subtypes, mediating different immune responses (Wang et al., 2015; Wang et al., 2018). Therefore, based on previous studies, we explored whether the Notch signaling pathway affects the development and differentiation of DCs, causing changes in the immune response of DCs sensitized with *E. granulosus* antigens, and then clarified whether it is involved in *E. granulosus* infection. By regulating the inhibition or activation of Notch signaling, the DC-mediated immune response may be affected and the course of the disease may be changed, which may be a new target or method to treat CE.

## 2 Materials and methods

### 2.1 Animal

6-8 weeks C57BL/6 wild-type mice were purchased from the Animal Laboratory Center at Ningxia Medical University in Yinchuan and 30 mice were bred in a specific pathogen-free facility in ventilated cage at room temperature (18-24°C).

### 2.2 Antigens of *E. granulosus*

Recombinant proteins Eg.ferritin and Eg.mMDH were expressed by Eg.ferritin/pET28a/BL21 and Eg.mMDH/pET28a/BL21 preserved in our laboratory and were purified using a Ni ^2+^affinity chromatography column (Roche, Basel, Switzerland), followed by quantitation and sterilization with a 0.45 μm filter (Millipore, Billerica,MA,USA), as described previously (Wang et al., 2018).

### 2.3 Bone marrow-derived dendritic cell (BMDC) culture *in vitro*

C57/BL/6 mice (6−8 weeks) mice were sacrificed and monocytes of bone marrow were obtained, as previously reported (Wang et al., 2015). Monocytes were counted and cultured for 7 days at 37°C, 5% CO_2_ incubator in 12-well plates in 1 ml RPMI1640 medium containing 10% fetal bovine serum (FBS)(Hyclone laboratories, Inc, Logan, UT,USA.) and 2 mM/L glutamine (Sigma-Aldrich, Saint Louis, MO, USA), 50 µM β-mercaptoethanol (Sigma-Aldrich, Saint Louis, MO, USA), 1% penicillin and streptomycin (Gibco Invitrogen, Carlsbad, CA, USA), supplemented with GM-CSF (10 ng/mL) and IL-4 (5 ng/mL) (R&D, Emeryville, CA,USA). The culture medium was changed at day 3 and 5, at day 7 the non-adhere cells were harvested, washed in PBS for twice and counted for following treatment.

### 2.4 DC treatment

#### 2.4.1 DC treated with DAPT

BMDCs were cultured with or without 50 µM DAPT (γ-secretase inhibitor) (Sigma-Aldrich, Saint Louis, MO,USA) on days 0, 3, and 5; on day 7 the cells were collected and suspended in 10% FBS medium without GM-CSF and IL-4. The cells were inoculated in a 24-well culture plate at a concentration of 1×10^6^ cells/mL per well. After 2 h of treatment with or without DAPT, 1 µg/mL Eg.ferritin or 1 µg/mL Eg.mMDH was added, followed by culture at 37°C, 5% CO_2_ for 20 h. Cells were collected for detection after centrifugation at 1200 rpm for 10 min at room temperature. The control was treated with dimethyl sulfoxide (DMSO) (Sigma-Aldrich, Saint Louis, MO,USA) at a final concentration of 0.25%.

#### 2.4.2 DC treated with Jagged1

On day 7, cultured cells were collected, resuspended, and inoculated as described above. The cells were divided into different groups according to their respective agents: (1) with and without addition of 1 µg/mL of Jagged1 (R&D, Emeryville, CA, USA), 1 µg/mL Eg.ferritin and 1 µg/mL Eg.mMDH respectively. (2) After 2 h of DAPT treatment, 1 µg/mL Jagged1 + 1 µg/mL Eg.ferritin 1, and 1 µg/mL Jagged1 + 1 µg/mL Eg.mMDH respectively, were added for 20 h. Cells were collected for detection by centrifugation at 1200 rpm for 10 min at room temperature.

### 2.5 Transmission electron microscopy (TEM)

Cells (3×10^6^) were treated with or without DAPT for 20 h and harvested, washed with PBS, fixed in 2.5% glutaraldehyde for 2 h, post-fixed with 1% osmium tetroxide (OsO4) for 1 h, dehydrated using gradient ethanol (30%, 50%, 70% and 90% 10 min 1 time, 100% thrice, 10min/time, and embedded in Epon 618 at 37°C for 6 h, 42°C for 18 h and 65°C for 18 h. Semi-thin and ultrathin sections of each sample were prepared, stained as described previously (Wang et al., 2015), and images were observed and captured using TEM (H-7650)(Hitachi Ltd., Tokyo, Japan).

### 2.6 Scanning electron microscopy (SEM)

On day 7 of DC culture, DCs from each group were collected, mixed, and treated with the pretreatment agent 50 µm DAPT. After 2 h, stimulants (Eg.ferritin and Eg.mMDH) were added. Cells (2×10^5^) taken from each group were inoculated on a cover slide in a 24-well culture plate and cultured for 20 h. The cells were washed thrice with PBS, fixed with 2.5% glutaraldehyde for 1 h, washed thrice with PBS, and dehydrated with ethanol. The samples were incubated in tert-butyl alcohol, vacuum dried, sputter-coated with a platinum film, and observed under SEM (S-3400N)(Hitachi Ltd., Tokyo, Japan). One hundred cells from each group were randomly counted and compared with the number of cells with or without dendrites. Dendrites < 3 were counted as negative (without dendrites). Twenty cells with dendrites were selected and counted, and the number of dendrites in each cell was analyzed. The experiments were conducted thrice.

### 2.7 Flow cytometry

#### 2.7.1 Surface marker expression of DCs

DCs (2×10^5^) were collected from each group and washed twice with PBS. Each sample was divided into two tubes containing anti-mouse CD11C-PE, anti-mouse MHCII-PE-CY5, anti-mouse CD80-FITC, anti-CD11C-PE, anti-mouse CD86- FITC and anti-CD40-PE cy5(MultiSciences, Hangzhou, China), respectively, in flow cytometry solution. The cells were incubated at 4°C for 30 min in the dark and centrifuged at 1500 rpm for 10 min at 4°C. Finally, each sample with at least 1×10^4^ cells was analyzed by flow cytometry (BD Accuri C6) (Becton, Dickinson and Company, New York, NJ, USA). The experiment was performed four times.

#### 2.7.2 DC Maturation stages

The cells were collected in groups on day 8 and stained with antibodies against CD11c. The percentage of the CD11c^+^ cell population was detected using flow cytometry, and the R1 gate was set according to the control group. Based on the R1 gate, the G1 and G2 gates were set according to the size and particles of the cells with side scatter (SSC) and forward scatter (FSC), which can be calculated as the percentage of mature DCs (mDCs) and immature DCs (iDCs) respectively. SSC^High^ (G1) represents mDCs and SSC^low^ (G2) represents iDCs. These data were analyzed using Flow J software. The method were described in the literature (Wang et al., 2009).

### 2.8 DC migration detection

On day 7, DCs were collected for DAPT treatment or no treatment for 2 h and exposed to Eg.ferritin or Eg.mMDH for 20 h, washed twice with RPMI 1640, and incubated with carboxyfluorescein diacetate succinimidyl ester (CFSE) (Sigma-Aldrich, Saint Louis, MO,USA) to a final concentration of 2.5 µ M/L at 37°C for 15 min in the dark, followed by incubation with pre-cooled 10% FBS for 5 min to stop the reaction. The cells were centrifuged at 1200 rpm for 10 min, washed twice with RPMI 1640 containing 10% FBS, mixed, counted, and the last 2×10^5^ cells were injected into the mouse footpad for 48 h. The mice were anesthetized with 7 μL/g 1% pentobarbital sodium (Sigma-Aldrich, Saint Louis, MO, USA) by intraperitoneal injection and the migration ability of DCs in mice was observed using the CRi Maestro *in vivo* imaging system and analyzed by Mi 5.3 stander Editor (Maestro EX)(Photometrics company, Tucson, AZ,USA). The experiment was repeated thrice.

### 2.9 Purification of CD4^+^ T cells

The spleen was removed aseptically and filtered through a 200-mesh filter. The spleen cell suspension was collected by adding erythrocyte lysate (Solarbio life Sciences, Beijing, China), followed by centrifugation for 10 min to obtain CD4^+^T cells for sorting according to Miltenyi Biotec CD4^+^ positive magnetic beads instructions. CFSE was added at a ratio of 1×10^7^ cells to a final concentration of 2.5−5μM/L, incubated for 15 min at 37°C, and the reaction was terminated with pre-cooled 10% FBS. After two washings with RPMI 1640 containing 10% FBS, cells were finally diluted to the desired concentration, avoiding light throughout the process.

### 2.10 Allogeneic mixed lymphocyte culture

On day 8, 2×10^5^ DCs (including control, Eg.ferritin, Eg.mMDH, DAPT+control, DAPT+Eg.ferritin, and DAPT+Eg.mMDH groups) were treated with final concentration 25μg/mL of mitomycin C (Merck & Co., Inc.,Kenilworth, IL, USA) for 30 min at 37°C as stimulated cells and CD4^+^T cells as effector cells, and mixed culture was performed at a ratio of 1:10 of DCs: CD4^+^ T. The wells were incubated for 120 h at 37°C in 5% CO_2_, and analyzed using flow cytometry. The proliferative ability of T cells was reflected by the green fluorescence intensity of the proliferation peak.

### 2.11 Quantitative real time RT- PCR (qRT-PCR)

Total RNA was extracted from BMDCs of different groups using TRIzol reagent (Invitrogen, Carlsbad, CA, USA), according to the manufacturer’s specifications. Total RNA was reverse-transcribed into cDNAs using the Revert Aid First Strand cDNA Synthesis Kit (Thermo Fisher Scientific (China) ltd, Shanghai, China). PCR was performed on a StepOnePlus™ Real-Time PCR instrument, according to the specifications indicated in Bestar™qPCR Master Mix (SYBR Green) (DBI Bioscience, Ludwigshafen, Germany). The PCR was operated under conditions of: 95°C for 2 min, followed by 40 cycles of denaturation at 95°C for 10 s, renaturation at 55°C for 30 s, and extension at 72°C for 30 s. The relative gene expression was calculated using the 2^−ΔΔCt^ method. Specific primer sequences were synthesized by the Sheng Gong Biology Company and are listed as follows in **Table 1**.

**Table 1:** Specific primer sequences.

Name	Up and down primer sequence	Length
Notch1	up:5′-CAGCTTGCACAACCAGACAGAC-3′ down:5′-ACGGAGTACGGCCCATGTT-3′	140 bp
Notch2	up: 5′-AGCAGGAGCAGGAGGTGATA-3′ down: 5′-TGGGCGTTTCTTGGACTCTC-3′	193 bp
Notch3	up: 5′-GACTGCTCACTGAACGTGGA-3′ down: 5′ -CACACCGGCTGTTGTTGAAG-3’	82 bp
Hes1	up:5′-AGAAAGATAGCTCCCGGCAT-3′ down:5′-TATTTCCCCAACACGCTCGG-3′	133 bp
Delta-4	up:5′- TTCCAGGCAACCTTCTCCGA-3′ down:5′-ACTGCCGCTATTCTTGTCCC-3′	102 bp
Jagged1	up: 5′-AGCTCACTTATTGCTGCGGT -3′ down: 5′-CCGCTTCCTTACACACCAGT-3′	153 bp
ß-actin	up:5’- CATCCGTAAAGACCTCTATGCC -3’ down:5’- ATGGAGCCACCGATCCACA-3’	171 bp

### 2.12 Cytokine assay

On day 8, DCs were collected from each group and centrifuged at 1200 rpm for 10 min, the supernatant was obtained, and the levels of IL-1β, IL-4, IL-6, IL-10, IFN-γ, TNF-α, and IL-12p70 were measured according to the instruction of Th1/Th2/Th9/Th22 cytokines 17-Plex Mouse ProcartaPlex™ Panel assay kit (Invitrogen, Carlsbad, CA,USA), using Luminex xMAP technology. Each sample was set to three replicates.

### 2.13 Statistical analysis

All data were analyzed using GraphPad Prism software (version 8.0) and are represented as mean ± standard error of the mean (SEM). The two-tailed t-test and one-way analysis of variance (ANOVA) were used to compare the groups. A *P* value less than 0.05 indicates a statistical difference between the two groups.

## 3 Result

### 3.1 Effect of DAPT on monocyte development at different times

**Figure 1:**
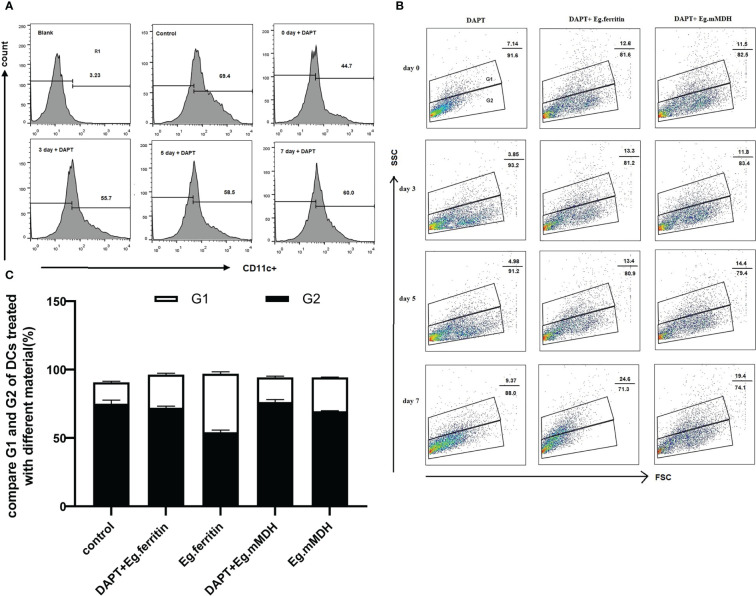
DAPT influences Monocyte development and DCs differentiation. Monocyte of bone marrow were treated with 50 µM DAPT on days 0, 3, 5 and 7 respectively and then DCs were collected and inoculated with the 1×10^6^ cells/mL in per well, exposed or did not exposed to 1 µg/mLEg.ferritin/Eg.mMDH for 20 h and DCs was treated with DMSO as control group. DCs of different groups were stained with antibody CD11c- PE and detected by flow cytometry. **(A)** R1 gate was set by control group, DAPT influences the development of monocyte to DCs were measured by the percentage of CD11c^+^ cell population. **(B)** Base on it, G1 and G2 gate were set according to side scatter(SSC) high and low, G1 represent the percentage of mDCs and G2 represent iDCs. **(C)** The effect of *E.granulosus* antigen or DAPT plus *E.granulosus* antigen on DCs differentiation were compared on day 7 (black bar represent G2 and white bar represent G1). The data were from four times experiments.

We observed the polarization of monocytes treated with 50 μM DAPT on days 0, 3, 5, and 7 through the detection of the CD11c^+^ expression cell population using flow cytometry, and the R1 gate was set. It was found that DAPT seriously impaired monocyte development into DCs at all time points compared with the control group, especially for day 0, when the percentage of DCs decreased to 44.7% (*P*<0.01), and the severity and monocyte count of culture time had a negative correlation. No distinct difference was observed between days 5 and 7 (**Figure 1A**). For consistency, on day 7, we selected DAPT to treat BMDCs for 2 h in later experiments. To determine the influence of DAPT on the differentiation of BMDCs, iDCs and mDCs were measured as the percentage of SSC^high^ and SSC^low^ cell populations. G1 was the side scatter (SSC)^high^ subgroup, representing mDCs, and G2 was the SSC^low^ cell subgroup, mainly iDCs. In addition, on day 7, different *E.granulosus* antigens were added to stimulate BMDCs that were pretreated with DAPT at different times to determine the percentage of iDCs and mDCs. In the DAPT-treated group, the percentage of differentiation of DCs to mDCs was much lower, especially on day 3, with only 3.85%, and the percentage of mDCs slightly increased on day 7. For the DAPT+ Eg.ferritin and DAPT+Eg.mMDH groups, there were no remarkable changes on days 0, 3, and 5, but the percentage of mDCs increased significantly on day 7 and Eg.ferritin stimulated BMDCs pretreated with DAPT increased mDCs by up to 24.6% (**Figure 1B**). This indicated that with the extension of DC culture time, the ability of DAPT to inhibit DC differentiation was weakened, and the ability of DCs to transform into mature cells under the action of antigens was gradually enhanced. However, on day 7, Eg.ferritin or Eg.mMDH stimulated BMDCs to produce 40.1% or 25.2% mDCs (the data were not shown), higher than the DAPT+Eg.ferritin and DAPT+Eg.mMDH groups, particularly for Eg.ferritin (*P*<0.05) (**Figure 1C**). These results showed that when the Notch signaling pathway was blocked, the effect of Eg.ferritin on DC function was significantly inhibited and that Eg.mMDH influence on DC differentiation was minimally impaired.

### 3.2 Effect of DAPT treatment on cytokine levels at different times

**Figure 2:**
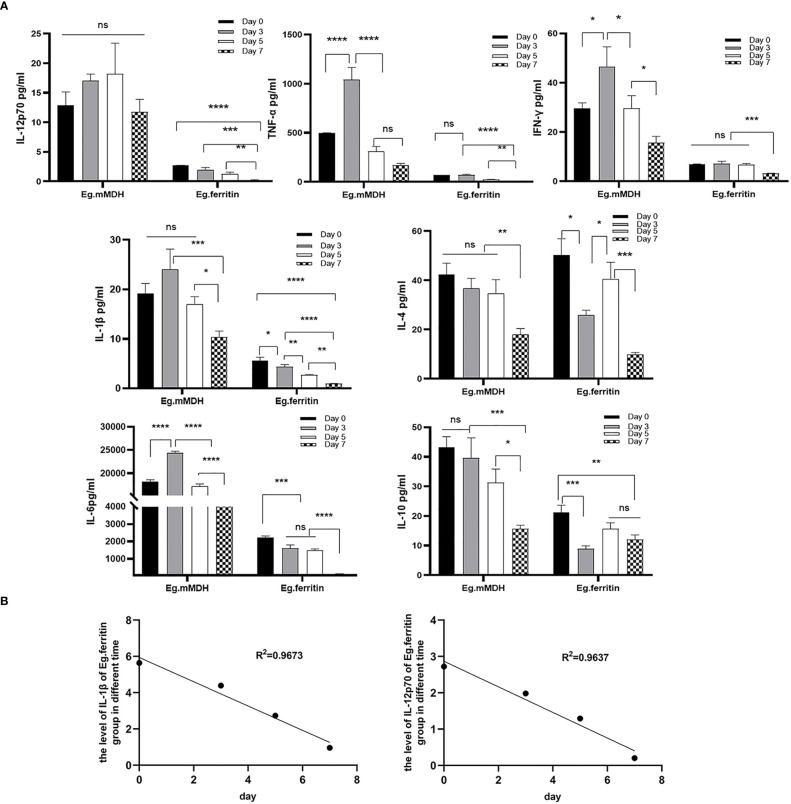
The level of cytokines of supernatant in different groups. Monocyte of bone marrow were treated with 50 µM DAPT on day 0, 3, 5 and 7, on day 7, DCs were collected and inoculated with the 1×10^6^ cells/mL in per well, then added 1 µg/mL Eg.ferritin or 1 µg/mL Eg.mMDH for 20 h. The supernatant of DC in different groups were obtained, **(A)** the level of IL-12p70, IL-1β, TNF-α, IFN-γ, IL-4, IL-6 and IL-10 cytokines were assayed. **(B)** The correlation between the expression levels of cytokines and the time of DAPT treated DCs was shown by linear regression. In the experiment, triple wells were set up for each sample, and one-way analysis of variance was used for data statistics. ****P<0.0001, ***P<0.001, **P<0.01, *P<0.05, ns means no statistical significance.

On day 7, different *E.granulosus* antigens were added to stimulate BMDCs that were pretreated with DAPT at different times. The supernatants of different groups were collected on day 8, and the levels of IL-4, IL-10, IL-6, IL-12p70, TNF-α, IL-1β, and IFN-γ were detected. The results showed an overall trend of the expression of various factors in the supernatant of the DCs of the Eg.mMDH group treated with DAPT being significantly higher than that of the Eg.ferritin group at all time points except for IL-4 (*P*>0.05). The levels of various cytokines in the Eg.mMDH group did not correlate with the time of DAPT treatment of DCs, except for the concentration of IL-12p70, which was not statistically different at each time point, whereas the expression of all other factors increased on day 3 and gradually decreased on days 5 and 7. However, TNF-α decreased significantly on day 5 (*P*<0.0001) with the levels of other cytokines being the lowest on day 7 (**Figure 2A**). Regarding the Eg.ferritin group, the levels of IL-12p70, TNF-α, IFN-γ, IL-1βand IL-6 decreased significantly with the time delay of the effect of DAPT on cultured DCs (*P*<0.0001), and there was a negative correlation between IL-12p70 and IL-1βwith the time of effect (R^2^ > 0.96, **Figure 2B**). The levels of IL-4 and IL-10 did not change significantly at different time points, decreasing on day 3, while both increased on day 5 and decreased again on day 7.

### 3.3 Surface markers of DCs stimulated with different materials

On day 7, DCs were collected and treated with or without 50 µM DAPT for 2 h, then exposed to Eg.ferritin or Eg.mMDH for 20 h, and the surface markers of DCs were detected. MHCII expression increased after treatment with Eg.ferritin, Eg.mMDH, DAPT+Eg.ferritin, and DAPT+Eg.mMDH and was higher than that in the control group (*P*<0.05). DCs exposed to Eg.ferritin, MHCII, and co-stimulatory molecules were all highly expressed, but DCs pretreated with DAPT +Eg.ferritin showed a sharp decrease (*P*<0.01). CD80 and CD86 expression decreased slightly in DAPT+Eg.mMDH compared to that in Eg.mMDH (*P*<0.05), and MCH II and CD40 expression did not differ significantly between the two groups (**Table 2**). These results indicate that DAPT affects DCs by inhibiting DC maturation, and reducing DC sensitization to Eg.ferritin and Eg.mMDH. In particular, the effect of ferritin on DC maturation was severely inhibited.

**Table 2:** The surface marker in DCs.

	MHCII	CD80	CD40	CD86
control (DMSO)	58.21 ± 3.76	20.92 ± 3.55	15.37 ± 1.42	29.34 ± 2.26
DAPT+Eg.ferritin	66.41 ± 8.32**a**	20.08 ± 2.98	28.01 ± 3.33**a**	24.31 ± 1.51
DAPT+Eg.mMDH	68.20 ± 6.14**a**	21.12 ± 1.74	19.23 ± 1.83	20.97 ± 2.25
Eg.ferritin	79.77 ± 4.95**ab**	58.57 ± 3.69**abc**	47.90 ± 3.97**abc**	52.01 ± 5.86**abc**
Eg.mMDH	69.87 ± 5.19**a**	27.74 ± 0.76**abc**	16.01 ± 0.54	27.94 ± 3.47**c**

On day 7, DCs of different groups were collected and treated with or without 50 µM DAPT for 2 h, then exposed to 1 µg/mLEg.ferritin or 1 µg/mLEg.mMDH for 20 h, detected the surface marker of DCs by flow cytometry. The results were expressed as the percentage of MHCII^+^, CD40^+^, CD80^+^, and CD86^+^ cells. Control group was DC treated with DMSO. The data were analyzed with the two-tailed t-test and presented as mean ± SEM of 4 independent experiments.

a refer to P<0.05, significantly different compared with control group.

b refer to P<0.05, significantly different compared with DAPT+Eg.ferritin group.

c refer to P<0.05, significantly different compared with DAPT+Eg.mMDH group.

### 3.4 The morphology of DCs treated with different materials

**Figure 3:**
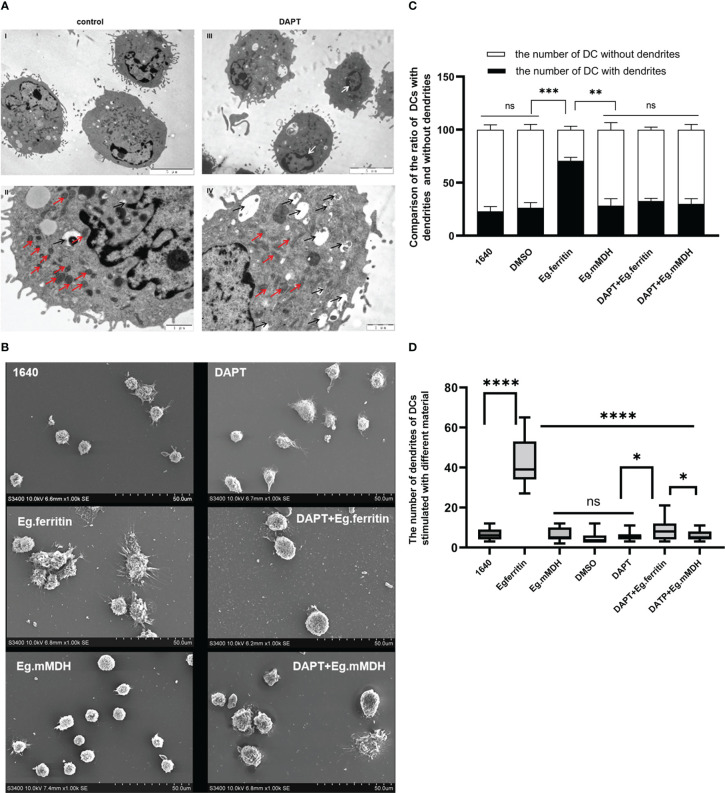
The morphology of DCs in different groups. **(A)** DCs were treated with or without 50 µM DAPT for 20 h, collected and prepared the samples to be observed by TEM. (blank arrow pointed autophagosome, red arrow showed mitochondria and white arrow showed that chromatin like crescent) DCs were pre-treated with or without 50 µM DAPT 2 h, and stimulated with or without 1 µg/mL Eg.ferritin or 1 µg/mL Eg.mMDH for 20 h. **(B)** The morphology of each group of DCs were observed by SEM. **(C)** One hundred cells from each group were randomly counted and the number of DCs with dendrites and the number of DCs without dendrite were compared in different groups (black part was the number of DCs dendrites and white part was the number of DCs without dendrite, ***P<0.001,**P<0.01,ns means no statistical significance.). **(D)** Twenty cells with dendrites were selected and the number of dendrites on DCs of each group were analyzed, ****P<0.0001, *P<0.05, ns means no statistical significance. The data were from thrice experiments.

The effect of DAPT on the morphology of DCs was observed using electron microscopy. Under TEM 1000× magnification, it was shown that in the DAPT-treated DC group, the size of DCs was slightly increased compared with the control group, the burr-like protrusions on the cell surface were few and short, and the type and number of intracellular organelles were less than those in the control group (**Figures 3A-I, III**). Under 3000 × magnification, we found that the number of mitochondria in DCs treated with DAPT was significantly lower than that in the control group, with large size, blurred morphology, and no visible contours and mitochondrial cristae (**Figures 3A-II**). Numerous autophagosomes of different sizes appeared in the cells, the nucleus was retracted, and chromatin like a crescent along the nuclear membrane showed early apoptosis (**Figures 3A-V**), indicating that DAPT induces DC autophagy and apoptosis. The results of SEM also showed that the size of DC treated with DAPT was larger than that of the corresponding DCs in the group not treated with DAPT, the surface was smooth, and surface burr-like protrusions were rare, showing the morphology of semi-mature DCs. However, DCs stimulated directly with Eg.ferritin showed rough cell surfaces with a large number of wrinkles and protuberances of different lengths, a manifestation of mature DCs (**Figure 3B**). One hundred cells were randomly selected from each group of DCs and the cells with and without burrs on the cell surface were counted and statistically analyzed. The number of DCs with burrs in the Eg.ferritin group was significantly higher than that in the control (DMSO) group and in other groups (*P*<0.001). After DAPT treatment, the proportion of DCs containing burrs in the total number of cells in the Eg.ferritin group was significantly reduced (*P*<0.001). This suggests that Eg.ferritin has the ability to stimulate DC maturation, and if DCs were pretreated with DAPT, the ability of Eg.ferritin to stimulate DC maturation was inhibited. In the Eg.mMDH group, DC maturation was not well stimulated, and most of the DCs were immature cells without burrs on the surface; with DAPT pretreatment, DCs exposed to Eg.mMDH showed no significant change in cell morphology (**Figure 3C**). Twenty DCs that had burrs on the surface in each group were selected, the number of burrs in each DC was counted, and the average number of burrs in each group was calculated and compared. In the Eg.ferritin group the average number of burrs was significantly higher than in the other groups(*P<*0.0001); however, the average number of burrs was significantly decreased in the DAPT+Eg.ferritin group, but these averages were still higher than those in the control, DAPT+Eg.mMDH, and Eg.mMDH groups (*P*<0.05) (**Figure 3D**).

### 3.5 Migration capability assessment of DCs

**Figure 4:**
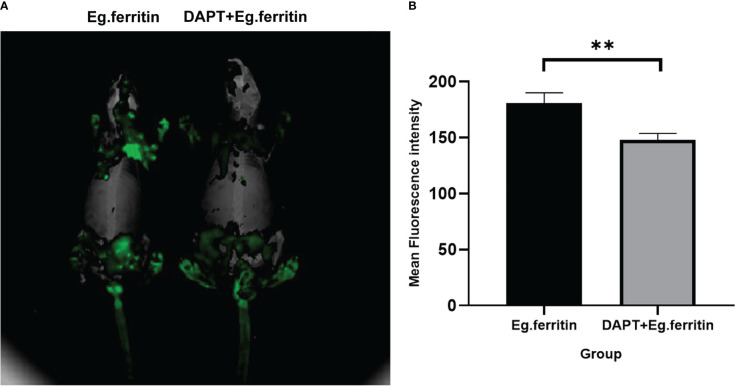
Assessment migration ability of DCs *in vivo*. DCs were treated with or without 50 µM DAPT for 2 h and exposed to 1 µg/mL Eg.ferritin or 1 µg/mL Eg.mMDH for 20 h, then the DCs of each group were collected and labeled with a final concentration of 2.5 µ M/L of CFSE, then injected into the footpads of mouse for 48h. The migration capability of DCs were detection by Cri Maestro *in vivo* imaging systems. **(A)** DCs migration ability observed (green fluorescence area of mouse means the amount of DCs migration) **(B)** Mean intensity of ROI comparison in different groups,** *P<*0.01.The data were from three independent experiments.

In the assessment of the migration capability of DCs in each group in mice, DCs treated with different materials were labeled with CFSE and injected into the footpads of mice, and migration ability was detected using the Cri Maestro animal imaging system. There was obvious green fluorescence in the groin and axillary lymph node areas of mice injected with Eg.ferritin-activated DCs, while in mice injected with DCs treated with DAPT+Eg.ferritin, fluorescence was observed only in the groin lymph node area (**Figure 4A**) and the mean intensity of the region of interest (ROI) was remarkably reduced (*P<*0.01) (**Figure 4B**). Because no obvious fluorescence was observed in the Eg.mMDH and DAPT+Eg.mMDH groups of mice, the data are not shown. These results indicate that the migration ability of DCs was attenuated by DAPT pretreatment and had a weak response to Eg.ferritin stimulation.

### 3.6 DCs treated with DAPT impaired CD4^+^T cell proliferation

**Figure 5:**
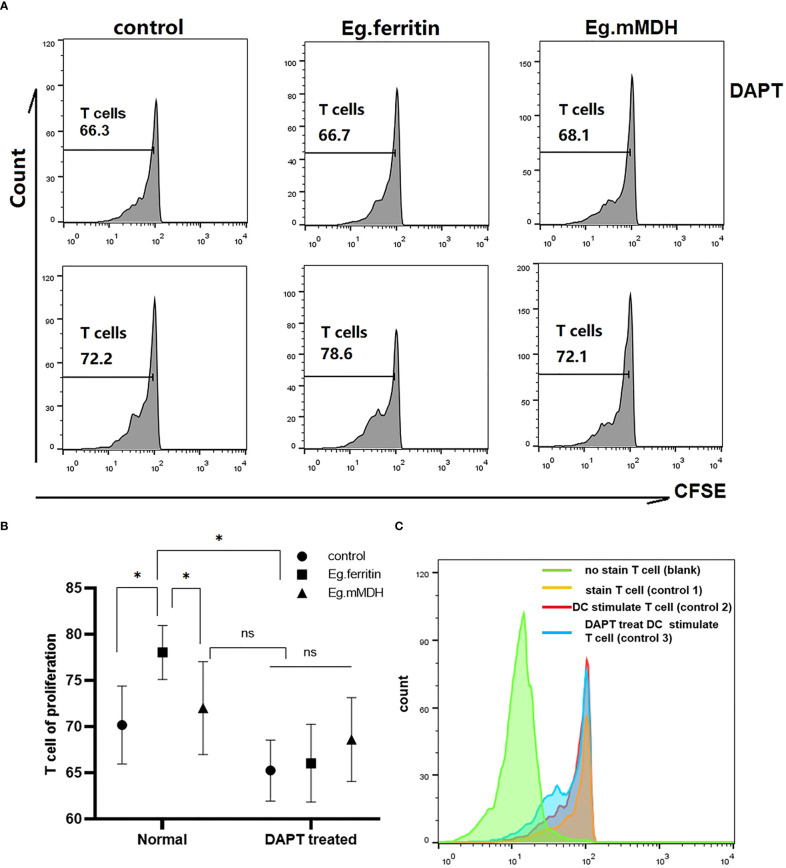
CD4^+^ T cell proliferation in different groups. DCs of each groups (control, Eg.ferritin, Eg.mMDH, DAPT+control, DAPT+Eg.ferritin, and DAPT+Eg.mMDH) as stimulator cells and CD4+T cells as effector cells, and mixed culture was performed at a ratio of 1:10 of DCs: CD4^+^ T for 120 h and 5 days later the cells were collected and analyzed by flow cytometry. **(A)** The proliferation of CD4^+^T cells coculture with DCs from different groups were reflected by the green fluorescence intensity. **(B)** And comparison the ability of CD4^+^ T cells proliferation in different groups (The data are means ± SEM from triplicate cultures, *P<0.05, ns means no statistical significance) **(C)** The detail of control groups in this experiment were shown (CD4^+^T cells without stained by CFSE as blank group, the CD4^+^T cells stained with CFSE as control group 1, the DC without treated by DAPT cocultured with CD4^+^T cells as control group 2 and the DC treated with DAPT cocultured with CD4^+^T cells as control group 3).

To evaluate the ability of DCs to induce T cell proliferation, all groups of DCs were collected as stimulator cells and cocultured with CD4^+^T cells as effector cells from the spleens of mice. We found that DAPT inhibited the ability of DCs to promote CD4^+^T cell proliferation. In control group 2, the proliferation ability of T cells was 72.2%, and the proliferation ability of T cells in control group 3 (DCs with DAPT treatment) decreased to 66.3% (**Figures 5A, B**). Both antigen-acting groups (Eg.ferritin and Eg.mMDH) were unable to promote T cell proliferation after DAPT treatment of DCs, with results significantly lower than those of the antigen-acting DCs alone especially for Eg.ferritin group (*P*<0.05)(**Figure 5C**). This indicated that DAPT impaired the antigen-presenting ability of DCs, causing no response in DC-mediated T-cell proliferation.

### 3.7 Expression of Hes1, Delta-4, Jagged1 and Notch molecules in mRNA of DCs with or without DAPT treatment

**Figure 6:**
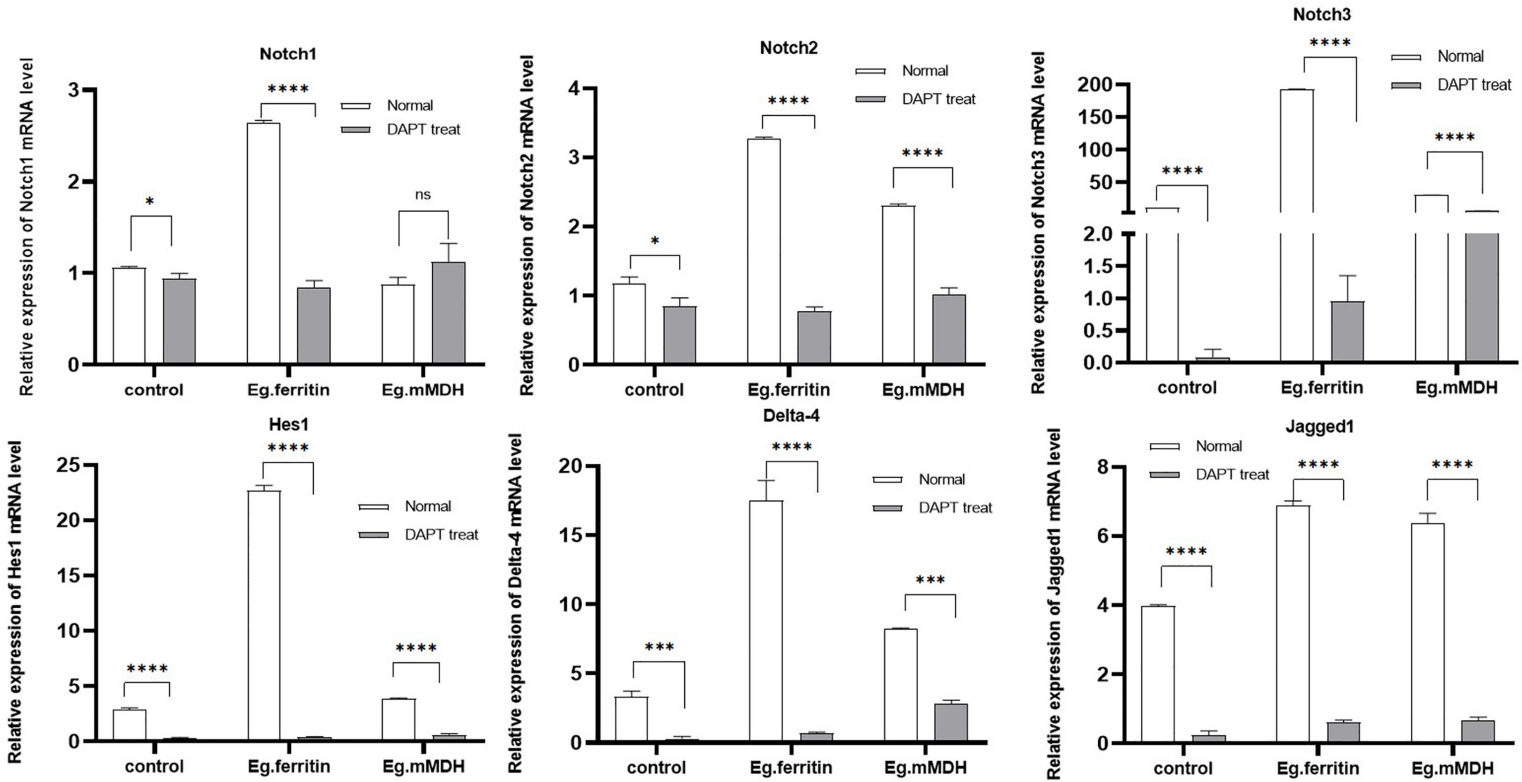
mRNA expression level of Notch, Hes1, Delta-4 and Jagged 1 in different groups of DCs. DCs were pre-treated with or without 50 µM DAPT for 2 h, then exposed to stimulants 1 µg/mL Eg. Ferritin or 1 µg/mL Eg.mMDH respectively for 20 h. Gray bars were DCs treated with DAPT followed by addition of Eg.ferritin or Eg. mMDH stimulants, control group was DC treated with DAPT. White bars were DCs stimulated with Eg.mMDH or Eg.ferritin alone, control group was DCs treated with 1640 RPIM. The data from three independent experiments and were analyzed by two-tailed t-test, *P<0.05, ***P<0.001, ****P<0.0001, ns means *P>*0.05.

To evaluate whether Notch signaling molecules affect the process of DC stimulation with Eg.ferritin and Eg.mMDH, the mRNA expression levels of Notch1, Notch2 and Notch3 receptors; Notch ligands Jagged1 and Delta-4; and Notch signaling pathway downstream target molecule Hes1 were measured by q-PCR. The results showed that Eg.ferritin could activate the expression of Notch receptors, ligands, and downstream target molecule Hes1, which was significantly higher than Eg.mMDH and the control group (*P*<0.0001), whereas Eg.mMDH only promoted the expression of Notch2, Jagged1, and Delta-4 compared with the control group (*P*<0.001). DAPT, an inhibitor of the Notch signaling pathway, remarkably inhibited the expression of Notch receptors and ligands and Hes1 in the control, and the expression of these molecules were reduced in the Eg.ferritin and Eg.mMDH groups to different degrees, especially in the Eg.ferritin group (*P*<0.0001). However, DAPT did not inhibit the expression of Notch1 in the Eg.mMDH group (*P*<0.0001), and did not inhibit Notch3 as much as the control group did (**Figure 6**). This suggests that Eg.ferritin can activate the Notch signaling pathway, whereas Eg.mMDH can only affect the expression of Notch2 receptors, Jagged1, and Delta-4 molecules.

### 3.8 Hes1, Delta-4, and Notch molecule mRNA expression in DCs treated with DAPT+ antigen or DAPT +Jagged1+ antigen

**Figure 7:**
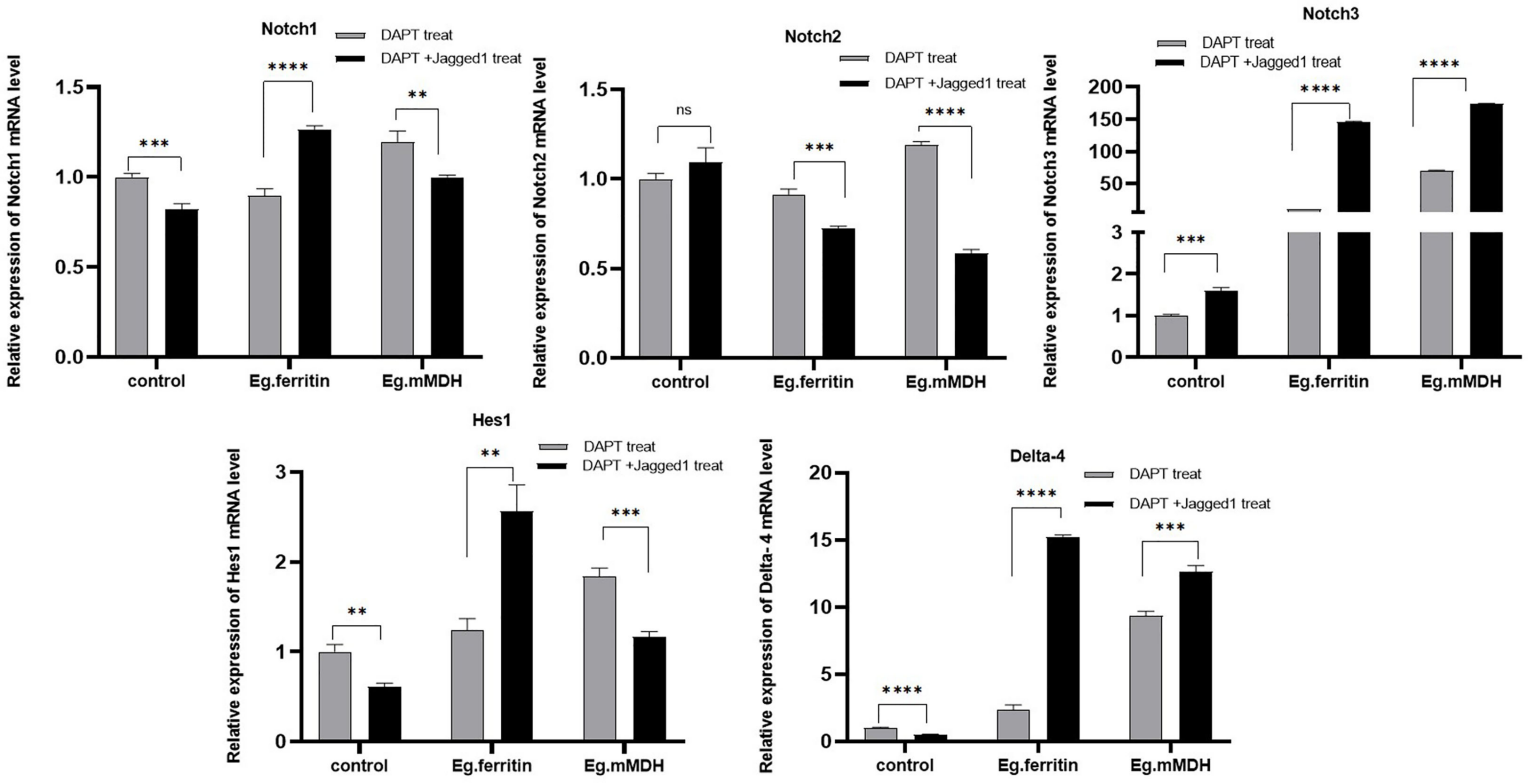
Activator Jagged1 influences mRNA expression level of Notch, Hes1, Delta-4 in different groups of DCs. DCs were pre-treated with 50 µM DAPT for 2 h, then added 1 µg/mL Jagged1 or without Jagged1 and at same time added stimulants 1 µg/mL Eg.ferritin or 1 µg/mL Eg.mMDH respectively. Gray bars were DCs treated with DAPT and followed by addition of Eg. ferritin or Eg. mMDH stimulants, control group was DCs treated with DAPT. Black bars were DCs treated with DAPT and added Jagged1 plus Eg.mMDH or Eg.ferritin, control group was DCs treated with DAPT+Jagged1. The data from three independent experiments and were analyzed by two-tailed t-test, **P<0.01, ***P<0.001, ****P<0.0001, ns means *P>*0.05.

To further verify the involvement of the Notch signaling pathway in the process of DC stimulation by *E.granulosus* antigens, DCs were treated with DAPT and Jagged1 was added as an activator of the Notch signaling pathway to antigens Eg.ferritin or Eg.mMDH. The expression levels of Notch receptors, ligands, and Hes1 were observed on day 7. The results showed that adding Jagged1 improved the expression of the Notch3 receptor in the control group (DAPT treatment), but further inhibited the mRNA expression of Notch1 and Delta-4(*P*<0.001). In the Eg.ferritin group, the expression of Notch1, Notch3, and Delta-4 was upregulated after adding Jagged1 (*P*<0.0001) and reached the previous level without DAPT treatment. Hes1 expression was also restored to some extent (*P*<0.01), but was still lower than that without DAPT treatment. This indicates that the Notch signaling pathway is involved in Eg.ferritin-sensitized DCs and may affect the function of DCs. In the Eg.mMDH group, Jagged1 significantly promoted the expression of Notch3 and Delta-4 (*P*<0.0001) but inhibited the expression of Notch2 and Hes1(*P*>0.05) (**Figure 7**). This indicates that only some Notch receptors are involved in the process of Eg.mMDH sensitization of DCs, and Jagged1 can only restore some of the molecules of the Notch signaling pathway.

## 4 Discussion

The Notch receptor undergoes protein hydrolysis in the presence of the γ-secretase complex, releasing Notch intracellular domain (NICD), which is transported *via* cellular endocytosis and membrane vesicles. When NICD translocates to the nucleus where it binds to the CSL or RBP-J to form a complex that undergoes a derepression effect, it activates the transcriptional activity of Notch target genes and promotes the expression of Hes downstream target genes (Kopan and Ilagan, 2009). Therefore, the γ-secretase complex is required for the activation of Notch signaling. The γ-secretase inhibitor DAPT, an inhibitor of the Notch signaling pathway (Wang et al., 2014) was used in our studies. BMDCs treated with DAPT mimicked the Notch signaling pathway blockage model *in vitro* and we observed the changes in DC and the response to the action of different antigens. Previous studies reported that the concentrations used for DAPT treatment of different cell types differed (Grottkau et al., 2009; Mathieu et al., 2013); DCs pretreated with an optimal concentration of 50 µM DAPT were used in our study (Shi-Yu et al., 2014). Most of the current literature reports studies of the effect of DAPT on immature DCs (Li et al., 2008). We consider that constructing a better Notch signaling pathway inhibition model by only observing the effect on immature DCs is not enough, because the inhibition of Notch signaling works throughout the development and differentiation of monocytes into DCs. Therefore, DAPT treatment of monocytes induced to DCs at different times (days 0, 3, 5, and 7) were observed separately to further clarify the effect of DAPT on DC development and differentiation. The CD11c^+^ cell percentage of total cells as DCs by flow cytometry revealed that DAPT had a significant inhibitory effect on DCs derived from monocytes cultured at the four time points and was negatively related to the number of days of DC development from monocytes (**Figure 1A**). This indicates that DAPT was able to arrest the development of monocytes into DCs, which led to a decrease in the number of DCs formed. TEM showed that DCs in the DAPT-treated group, with numerous autophagosomes in the cytoplasm and nuclei solidified into early apoptotic manifestations (**Figure 3A**), which may be the cause of apoptosis of monocyte precursors under the continuous effect of DAPT (Palaga et al., 2013), which led to a decrease in the number of cells induced to differentiate into DCs. DCs at day 7 of DAPT treatment showed an increase in CD11c^+^ expression, which may be because most of the monocytes at day 7 had developed into DCs, and the number of DCs was less affected by DAPT. Eg.ferritin- and Eg.mMDH-stimulated DCs pretreated with DAPT at different time points, showed that the percentage of DCs differentiated into mature DCs (G1) after antigen stimulation increased slowly with increasing days of culture and was higher in the DAPT group with Eg.ferritin (**Figure 1B**), but was still significantly lower than that of Eg.ferritin alone. Presumably, DAPT inhibits or closes some of the receptors for antigen uptake on the surface of DCs, showing a low response to Eg.ferritin. The differentiation of DCs in the Eg.mMDH group and in the DAPT+Eg.mMDH group was not significant (**Figure 1C**), suggesting that they may rarely be affected by DAPT. On this basis, we further investigated the DCs on day 7 of DAPT treatment; SEM showed that the cell surface was smooth with no or few protrusions, but the size was significantly larger than the control group, showing the characteristics of semi-mature morphology (Riganò et al., 2007) (**Figure 3B**), and the expression of mature molecules MHCII, CD80, CD86 and CD40 on the surface of DCs was low (**Table 1**). The ability of DC-mediated T cell proliferation was remarkably impaired in all groups with DCs pretreated with DAPT compared to groups with DCs not treated with DAPT (**Figure 5**). This phenomenon was further verified by the *in vivo* DC migration ability assay. The Cri Maestro *in vivo* imaging systems showed that DCs stimulated by Eg.ferritin alone could migrate to inguinal and axillary lymph node areas in mice; however, when DCs were pretreated with DAPT, the Eg.ferritin-stimulated group showed a weak DC migration ability in mice (**Figure 4**). This suggests that DAPT reduced the migration ability of DCs by blocking the Notch signaling pathway and preventing their differentiation into mature DCs. These results are consistent with the findings of Wang et al. (Wang et al., 2009), who used a conditional knockout of Notch signaling deletion on DCs in RBP-J gene mice. From the above results, we speculate that DAPT can inhibit the early development of monocytes to DCs, and as monocytes induce the development of DCs for a longer period, DCs become more functional and can respond to external antigenic stimuli. At this time, the effect of DAPT on DC development decreases, but significantly inhibits DC maturation. In contrast, the shorter the time of DC development induced by monocytes, the greater the DAPT damage to the function of DCs. Although some monocytes can develop into DCs, these DCs may be inactive due to incomplete development or abnormal function, so they show no response to effective antigens and cannot be effectively differentiated to mature.

To further understand the changes in the Notch signaling pathway-related molecules under the blockage of DAPT, the mRNA expression of Notch1, Notch2 and Notch3, Jagged1, Delta-4, and Hes1 was examined in each group of DCs. DCs in both the Eg.ferritin and LPS groups (data not shown) expressed Notch1-3, Hes1, and Delta-4 at significantly higher levels than those in Eg.mMDH and control groups, whereas DAPT treatment of DCs caused a dramatic decrease in the expression of various Notch receptors, ligands, and Hes1 to the level of the control group (**Figure 6**) and mainly inhibited the secretion of pro-inflammatory factors IL-1ß, IL-6, TNF-α, IL-12p70, and IFN-γ (**Figure 2**). These findings are similar to those of Tsao (Tsao et al., 2011). We speculate that Eg.ferritin may directly activate the Notch signaling pathway similar to LPS. Its effect on DCs (Ito et al., 2009) can highly express Delta-4 and Jagged1, and through DAPT can severely hinder the differentiation and function of DCs inhibiting the proliferation of Th1-type cells (Dua et al., 2019), thus affecting the occurrence of a positive immune response, attenuating the secretion of inflammatory factors (Hildebrand et al., 2018), and relieving the inflammatory response of the organism. These factors may contribute to pathogen invasion and aggravate the severity of infection. This suggests that Notch signaling contributes effectively to the response mainly through the modulation of DC function in activating an effective acquired immune response (Neal et al., 2017); thus, blocking the Notch pathway could affect DC-mediated immunity by Eg.ferritin sensitization to control *E. granulosus* infection.

To further verify this speculation, Jagged1 was chosen as an activator of the Notch signaling pathway (Weijzen et al., 2002). Jagged1, as a ligand for various Notch receptors, including Notch1, Notch2 and Notch3, activates Notch signaling through RBP-J, and is abundantly expressed on the surface of antigen-presenting cells, DCs, B cells, and macrophages (Shimizu et al., 1999). In this study, we used the Jagged1 peptide to treat BMDCs to simulate Notch signaling activation *in vitro*. Observation of DCs pretreated with Jagged1 and subjected to Eg.ferritin did not reveal DCs that were differentiated to mature morphology with increased surface protrusions compared to DCs stimulated with Eg.ferritin alone (data not shown). In back-complementation experiments, the addition of Jagged1 to DAPT-treated DCs slightly restored the Notch3 expression, while the expression of other molecules continued to decrease. However, after stimulation with the antigen Eg.ferritin, the Notch signaling pathway was activated, and the expression of Notch1, Delta-4, and Hes1 was significantly increased (**Figure 7**). This indicates that Eg.ferritin activates the Notch1/Jagged1 signal and activates higher expression of Notch1, which plays a major role in DC maturation and active Th1 response in parasitic infections (Auderset et al., 2012). Therefore, Notch1 activation may be a viable strategy for *in vivo* and *ex vivo* immunomodulation in applications such as DC-based tumor vaccines or vaccines against infectious agents or toxins (Weijzen et al., 2002), which verified that Eg.ferritin is a potential vaccine against cystic hydatid infection, which is consistent with our previous report (Wang et al., 2015). For the Eg.mMDH group, the addition of Jagged1 and Eg.mMDH antigen did not activate the expression of various molecules of the Notch signaling pathway, except for a mild upregulation of Notch3 and Delta-4 expression. The response of Eg.mMDH-exposed DCs may play an independent role in the RBP-J signaling pathway (Dontje et al., 2006).

In summary, Notch signaling is involved in Eg.ferritin-stimulated DC-induced immune responses by modulating DC development and maturation. Therefore, activation or inhibition of Notch signaling could regulate the immune response mediated by Eg.ferritin-stimulated DCs. Regulating the Notch pathway, or targeting Notch ligands may provide a new perspective on the control of *E. granulosus* infection (Chandrakar et al., 2021) and inflammatory responses (Castro et al., 2021). However, this research has only been done *in vitro*, which has some limitations and needs further *in vivo* experiments for validation.

## 5 Conclusion

Form the study, it indicates that Notch signaling is involved in Eg.ferritin-stimulated DC-induced immune responses by modulating DC development and maturation. But, Notch signaling is not involved in the response of Eg.mMDH-exposed DCs.

## Data availability statement

The original contributions presented in the study are included in the article/supplementary material. Further inquiries can be directed to the corresponding author.

## Ethics statement

All mouse experiments were approved by the Ningxia Medical University Institutional Review Committee (permit number: 2021-N135) and were performed in strict accordance with national and institutional guidelines.

## Author contributions

MW observed the photographs of microscope and conducted the data analyses, ZS and conducted all the animal experiments, FQ purified the protein and designed primer of genes, ZS and FQ cultured cells, JH and XM bred animal and prepared samples for flow cytometry. MW, ZS, and FQ wrote the manuscript. YW granted funding, conceived and designed the experiments, edited the manuscript. All authors contributed to the article and approved the submitted version.
